# *Vf*ODB: a comprehensive database of ESTs, EST-SSRs, mtSSRs, microRNA-target markers and genetic maps in *Vicia faba*

**DOI:** 10.1093/aobpla/plaa064

**Published:** 2020-11-29

**Authors:** Morad M Mokhtar, Ebtissam H A Hussein, Salah El-Din S El-Assal, Mohamed A M Atia

**Affiliations:** 1 Molecular Genetics and Genome Mapping Laboratory, Genome Mapping Department, Agricultural Genetic Engineering Research Institute (AGERI), Agricultural Research Center (ARC), Giza, Egypt; 2 Genetics Department, Faculty of Agriculture, Cairo University, Giza, Egypt

**Keywords:** Faba bean, genetic maps, molecular breeding, molecular markers, *Vicia faba*

## Abstract

Faba bean (*Vicia faba*) is an essential food and fodder legume crop worldwide due to its high content of proteins and fibres. Molecular markers tools represent an invaluable tool for faba bean breeders towards rapid crop improvement. Although there have historically been few *V. faba* genome resources available, several transcriptomes and mitochondrial genome sequence data have been released. These data in addition to previously developed genetic linkage maps represent a great resource for developing functional markers and maps that can accelerate the faba bean breeding programmes. Here, we present the *Vicia faba* Omics database (*Vf*ODB) as a comprehensive database integrating germplasm information, expressed sequence tags (ESTs), expressed sequence tags-simple sequence repeats (EST-SSRs), and mitochondrial-simple sequence repeats (mtSSRs), microRNA-target markers and genetic maps in faba bean. In addition, KEGG pathway-based markers and functional maps are integrated as a novel class of annotation-based markers/maps. Collectively, we developed 31 536 EST markers, 9071 EST-SSR markers and 3023 microRNA-target markers based on *V. faba* RefTrans V2 mining. By mapping 7940 EST and 2282 EST-SSR markers against the KEGG pathways database we successfully developed 107 functional maps. Also, 40 mtSSR markers were developed based on mitochondrial genome mining. On the data curation level, we retrieved 3461 markers representing 12 types of markers (CAPS, EST, EST-SSR, Gene marker, INDEL, Isozyme, ISSR, RAPD, SCAR, RGA, SNP and SSR), which mapped across 18 *V. faba* genetic linkage maps. *Vf*ODB provides two user-friendly tools to identify, classify SSR motifs and *in silico* amplify their targets. *Vf*ODB can serve as a powerful database and helpful platform for faba bean research community as well as breeders interested in Genomics-Assisted Breeding.

## Teaser

Here, we present the *Vicia faba* Omics database (*Vf*ODB) as a comprehensive database integrating 31 536 EST, 9071 EST-SSR and 3023 microRNA-target markers developed based on *V. faba* RefTrans V2 mining. By mapping 7940 EST and 2282 EST-SSR markers against the KEGG pathways database, we successfully developed 107 KEGG pathway-based maps. At the data curation level, we retrieved 3461 markers representing (CAPS, EST, EST-SSR, INDEL, ISSR, SCAR, RGA, SNP and SSR) markers, which mapped across 18 *V. faba* genetic linkage maps. *Vf*ODB can serve as a powerful database for the faba bean research community and breeders interested in Genomics-Assisted Breeding.

## Introduction

Faba bean also called broad beans (*Vicia faba*) is one of the most important food and fodder legume crops worldwide after pea, chickpea and lentil (FAOSTAT 2017). Its importance is due to the high levels of protein and fibres (i.e. 25–40 %), in addition to its contribution to agricultural sustainability through nitrogen fixation and soil-improvement capabilities (Rispail *et al.* 2010). Moreover, it has extra advantages over other legume crops as it can adapt and grow under limited irrigation (Al-Suhaibani 2009; Alderfasi and Alghamdi 2010), moderate salinity (Abdelhamid *et al.* 2010) and low temperatures (cool climates) conditions (Kaur *et al.* 2014).

The global production of *V. faba* is 4.84 million tonnes; it is led by China which produces ~37.2 % of the total worldwide crop, followed by Ethiopia (19.2 %), Australia (7.7 %), UK (6.2 %), Germany (3.9 %), France (3.9 %) and Egypt (2.3 %) (FAOSTAT 2017; http://faostat3.fao.org). Meanwhile, Egypt is ranked as the world’s largest importer of faba beans with an estimated production–consumption gap of about 73 % in 2014. Due to this huge gap, Egypt imports annually over half of the global imports (Ouda and Zohry 2017).

Faba bean is a diploid plant (2*n* = 12) with one of the largest genomes among legumes (~13 000 Mb). Its genome is 26× larger than the *Medicago truncatula* (as a model plant) and repetitive DNA represents over 85 % of its genome composition (Flavell *et al.* 1974; Sato *et al.* 2010; Khazaei *et al.* 2014).

During the last decade, many faba bean transcriptome data have been released; meanwhile, remarkably limited genomic DNA sequence data are deposited to public databases (Ray and Georges 2010; Kaur *et al.* 2012; Yang *et al.* 2012; Arun-Chinnappa and McCurdy 2015). The only *V. faba* genomic DNA sequences data set was reported by Yang *et al*. (2012), in which, Pyrosequencing of pooled genomic DNA of 247 accessions was performed to identify and develop microsatellites markers. To date, this obvious lack of publicly available genome resources in faba bean is mainly attributed to the intrinsic difficulties of decoding this giant genome (Torres *et al.* 2010; Kaur *et al.* 2014).

The above-mentioned limitation in genomic resources availability consequently affected the development of successful marker-assisted selection (MAS) breeding programmes in faba bean comparing to other legume species (Avila *et al.* 2004). Taking into consideration that the majority of economically important traits are controlled by multiple genes, limited success is expected through the implementation of traditional breeding methods. Therefore, the application of MAS strategies provides a potential solution to overcome such a problem, through the development of effective genetic markers and genetic linkage maps of markers controlling economic traits (Kaur *et al.* 2014).

On the other side, earlier mapping studies have been developed with the aid of random amplified polymorphic DNA (RAPD) and sequence-characterized amplified region (SCAR) markers (Gutierrez *et al.* 2007; Ellwood *et al.* 2008; Díaz-Ruiz *et al.* 2010). Later, the development of expressed sequence tags (ESTs), microsatellites or Single Sequence Repeats (SSRs), EST-SSRs and single nucleotide polymorphism (SNP) markers added a step forward to relatively enrich faba bean genetic studies and breeding (Zeid *et al.* 2009; Gong *et al.* 2010; Torres *et al.* 2010; Ma *et al.* 2011; Wang *et al.* 2011; Kaur *et al.* 2012; Suresh *et al.* 2013; Khazaei *et al.* 2015; Webb *et al.* 2016; Beier *et al.* 2017).

In an initial step in 2008, the ‘Vicia Toolbox’ website was released as an online hub aimed at gathering researchers and breeders who are interested in developing community resources and collaborative research with the goal of genetic improvement of faba bean (https://www.viciatoolbox.org/). Two years later, the Pulse Crop Database (PCD), formerly the Cool Season Food Legume Database (CSFL; https://www.pulsedb.org) was developed to translate the released genomics knowledge into the crop improvement framework of many legume crops such as pea, lentil, chickpea and faba bean.

The integration of curated and developed genomic, transcriptomic, gene-ontology and KEGG pathway data in an open-platform as species-specific database integrating various Omics data and germplasm information is an essential demand for faba bean researchers and breeders.

Here, we present the *Vicia faba* Omics database (*Vf*ODB) as a comprehensive hub of germplasm information, ESTs, EST-SSRs, mtSSRs, microRNAs markers and genetic maps in faba bean. Additionally, we developed a KEGG pathway-based markers database, as a novel class of annotation-based markers. We expect that *Vf*ODB will serve as a beneficial platform for researchers interested in Genomics-Assisted Breeding (GAB) in faba bean.

## Materials and Methods

### Data collection

At first, we downloaded the *V. faba* mitochondrial genome sequence and its annotation from the NCBI GenBank database (accession number; KC189947). Regarding the *V. faba* transcriptome, since many versions were available, the recent reference and enriched transcriptome version (CSFL *V. faba* RefTrans V2; which combines all published RNA-Seq and EST data sets to create a reference transcriptome) was downloaded in addition to its associated annotations from the Pulse Crop Database. Moreover, we also retrieved KEGG pathways, KEGG orthologs and Gene Ontology annotations of the *V. faba* Reference Transcriptome - version 2.

On the other hand, to curate all previously mapped DNA markers in *V. faba*, an extensive PubMed search was performed to obtain all relevant publications till October 2020, using different sets of keywords. Thereafter, each paper was carefully checked to collect all DNA marker information, including marker name, primer sequence, product size, marker position on the map, etc. Moreover, due to that, not all curated markers are annotated; we utilized several online tools to annotate these markers. Comprehensively, we curated all available faba bean germplasm information from both the Genebank Information System of the Genesys Database supported by CGIAR (https://www.genesys-pgr.org) as well as from the IPK Genebank at Gatersleben, Germany (https://gbis.ipk-gatersleben.de/) and finally listed, categorized and implemented in our *Vf*ODB database.

### Bio-data mining, curation and database construction

In this study, we divided the bio-data mining work into three main categories: 1) Mitochondrial-based markers, 2) Transcriptome-based markers, and 3) KEGG-based markers. In addition to, 4) Genetic maps-based markers data curation and annotation.

**Figure 1.**
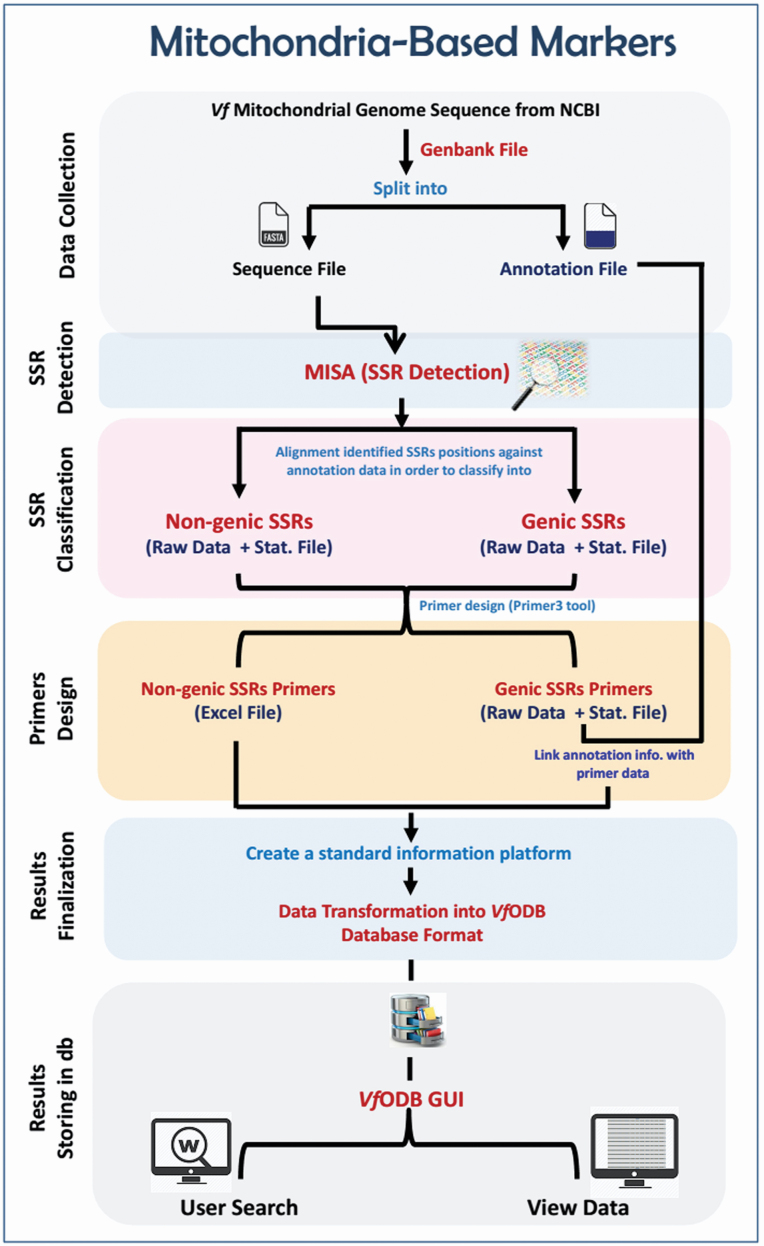
The workflow of mitochondrial-based markers development.

**1) For mitochondrial-based markers development**, the downloaded *V. faba* mitochondrial genome was analysed in terms of microsatellites identification, classification and marker development. The workflow of analysis was done in five main steps, as follows: (a) detection of SSR motifs on the genome-scale; (b) classifying identified SSR motifs into non-genic or genic according to their location within the genome; (c) designing SSR primers and developing markers; (d) organizing and integrating developed SSR markers as well as their associated information into the *Vf*ODB database; and (e) implementing all generated data sets into the *Vf*ODB web interface (Fig. 1).

Technically, the MISA (MIcroSAtellite identification) tool (Beier *et al.* 2017) was used to identify and localize all perfect SSR motifs as well as compound SSR motifs within the *V. faba* mitochondrial genome. For perfect motifs, the parameters were adjusted as following: mono- (≥10), di- (≥6), tri- (≥5), tetra- (≥4), penta- (≥3) and hexa-nucleotide (≥3). For compound motifs, the parameters were adjusted to identify motifs with ≥2 repeats interrupted by ≤100 bp. The obtained results were organized through developed in-house-Perl scripts and finally compared against the mitochondrial genome annotation to classify the SSR motifs into non-genic and genic motifs. Based on each identified motif coordinates within the mitochondrial genome sequence, a 200-bp flanking sequence of each motif was extracted instead of using the full genome sequence. Thereafter, SSR primers were designed for all classified SSR motifs using Primer3 software (Untergasser *et al.* 2012), and a unique marker ID was then assigned for each marker. Finally, the Genome Browser Tool (known as JBrowse) (Buels *et al.* 2016) was implemented in our *Vf*ODB database to manipulate and display the coordinates of SSR motifs/primers within the *V. faba* mitochondrial genome.

**Figure 2.**
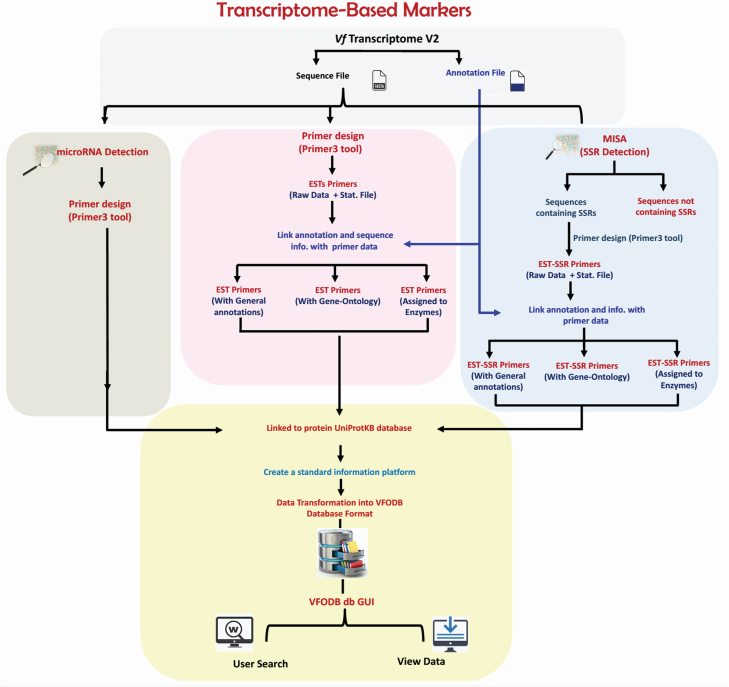
The workflow of transcriptome-based markers.

**2) For Transcriptome-based markers**, the downloaded *V. faba* reference transcriptome V2 was analysed for detecting three items: 1) transcriptome-wide EST markers, 2) microsatellites (EST-SSR markers development) and 3) microRNA-targets (Fig. 2).

For transcriptome-wide EST markers development, the *V. faba* RefTrans V2 (37 378 sequences) was subjected to Primer3 software in order to develop transcriptome-wide EST markers according to the following criteria: primer length 20 bp; melting temperature of 55 °C; product size range of 100–500 bp; and a 50 % G/C content (Mokhtar and Atia 2019). Later, the developed EST primers data were combined with their annotation information and classified into one of the following classes: 1) EST primers with general annotation, 2) EST primers with gene-ontology and 3) EST primers assigned to certain enzymes.

For EST-SSR markers development, the *V. faba* RefTrans V2 was analysed using the MISA tool to identify the sequences containing SSR motifs, which were consequently extracted and subjected to Primer3 software (Untergasser *et al.* 2012) to develop EST-SSR markers. These markers were subsequently combined with their annotation information and also classified into three classes: 1) EST-SSR primers with general annotation, 2) EST-SSR primers with gene-ontology and 3) EST-SSR primers assigned to certain enzymes.

MicroRNA-target markers were identified using the psRNATarget online tool (psRNATarget: A Plant Small RNA Target Analysis Server; http://plantgrn.noble.org/psRNATarget/home) to predict all plant microRNA families targets within the *V. faba* RefTrans V2. These targets were consequently extracted and subjected to Primer3 software to develop microRNA-targets based markers.

Finally, all developed markers (ESTs, EST-SSRs and microRNA-targets based markers) were linked to the JBrowse tool to display their coordinates within the sequences of *V. faba* RefTrans V2.

**Figure 3.**
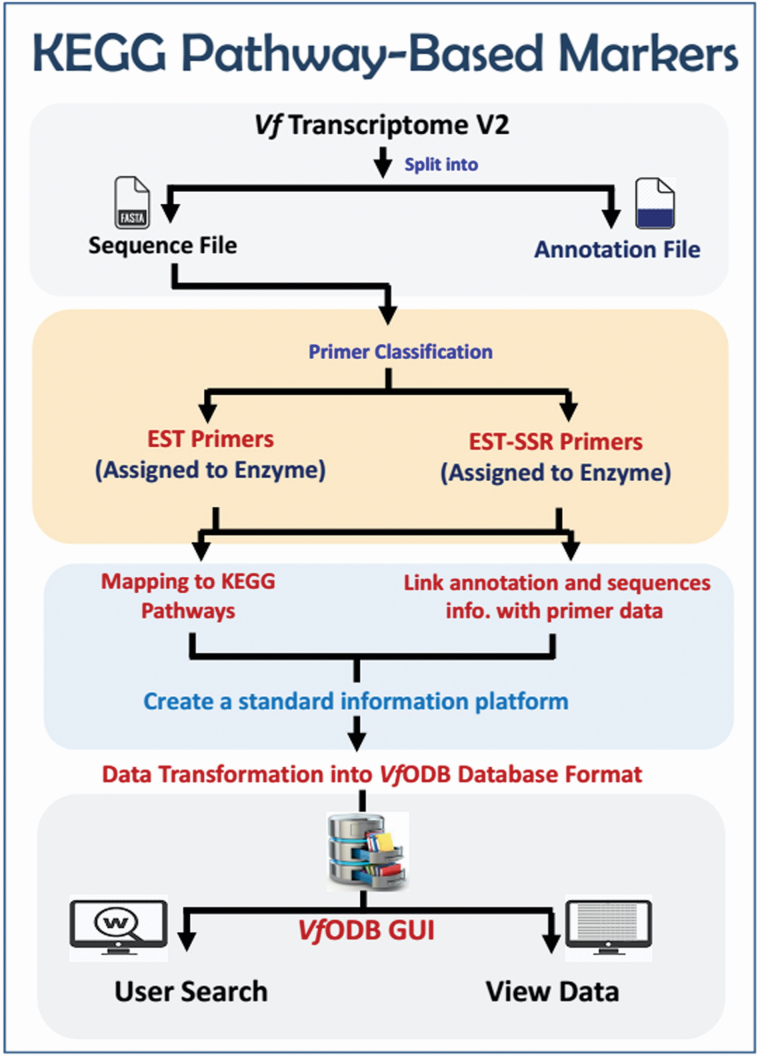
The workflow of KEGG pathway-based markers.

**3) For KEGG pathway-based markers**, the developed EST and EST-SSR markers (assigned to enzymes) were further mapped against the KEGG pathways database (https://www.genome.jp/kegg/pathway.html) to build *V. faba* functional maps (Fig. 3). Each map contains; Pathway ID, Pathway image, mapped enzymes ID (highlighted), mapped enzymes associated primers/markers, markers annotation information, markers coordinates (within RefTrans V2 and hyperlinked to the JBrowse viewer) and all other information related to this primer (Tm, GC%, Length, etc.). All generated maps were finalized in a user-friendly attractive form.

**Figure 4.**
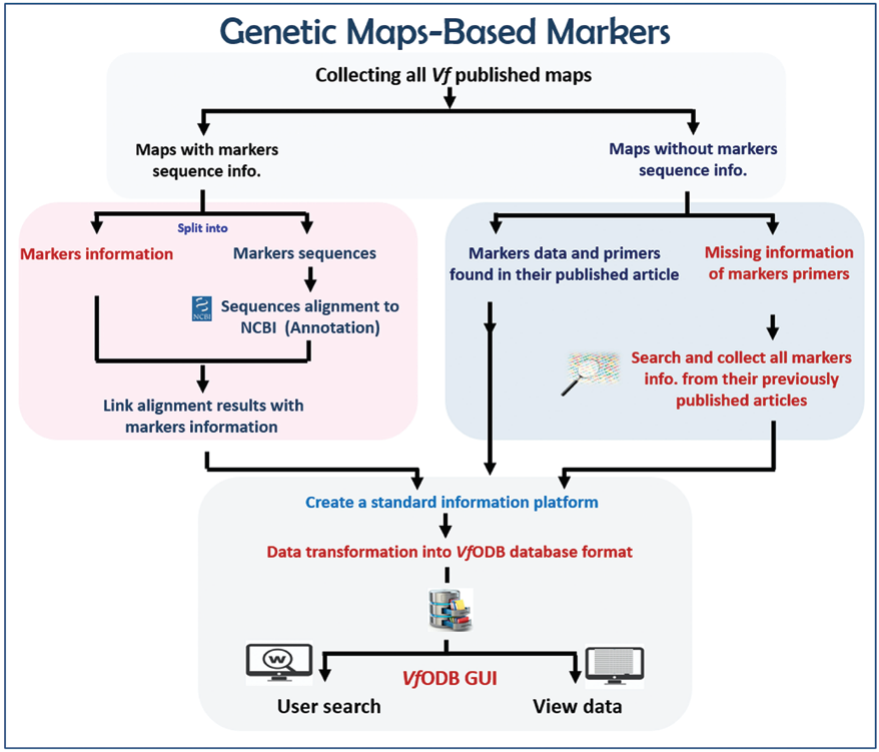
The workflow of genetic maps-based markers.

**4) For Genetic maps-based markers**, we downloaded all previously developed and published genetic linkage maps of *V. faba*. All available information of mapped markers on these maps were manually curated and categorized according to their type. In case that markers sequence was available such as SNP markers sequence, these sequences were further curated and aligned against the NCBI GenBank database by using blastx tool to determine its corresponding protein (Fig. 4).

Furthermore, all developed annotated markers belonging to different marker types (EST, EST-SSR and microRNA-target markers) were later combined with their functional information available at the UniPort knowledgebase (UniProtKB; https://www.uniprot.org/uniprot/). Finally, all generated and curated data sets of markers and functional maps were transformed to build a standard information platform for all marker classes and maps to be integrated into the *Vf*ODB SQL database. The *Vf*ODB database was implemented using a combination of Linux, Perl, PHP and MySQL applications platform. Additionally, the CSS, JavaScript script language and Hypertext Markup Language (HTML) were implemented to design a *Vf*ODB user-friendly web interface.

## Results

### *Vf*ODB interface

The *Vf*ODB database provides a modern web interface supported with multiple effective and powerful features including; exploring, downloading, searching and analysing tools. The *Vf*ODB website affords an effective navigation bar designed to facilitate users browsing across the different sections of the *Vf*ODB hub responsively and conveniently. The stored data in *Vf*ODB can be effortlessly browsed, accessed, searched and retrieved via seven interactive pages: Homepage, Transcriptome (JBrowse, EST markers, Validated ESTs, EST-SSR markers and Validated EST-SSR), Mitochondrial (JBrowse, Mitochondrial-SSR and Validated Mitochondrial-SSR), microRNA (JBrowse, Predicted microRNA-targets and markers), Molecular Maps (Genetic Maps and KEGG Pathway-based Maps), Tools (MISA and *in silico* PCR) and Germplasm. Users can browse the above-mentioned sections of the *Vf*ODB database in a very simple way through choosing any item from the drop-down menus located in the main navigation bar.

The Homepage of *Vf*ODB provides a simple introduction and images about faba bean and its economic importance, as well as offering an overview of the main workflows used to develop the *Vf*ODB database and highlighting general statistics of obtained results within each section.

For the Transcriptome drop-down menu, searches are divided into five separate pages according to the molecular marker type/class. Pages of ‘EST Markers’, ‘EST-SSR Markers’ and ‘Validate EST or EST-SSR Primers’ provide users with different utilities presented in a sub-pages style and include: 1) EST or EST-SSR markers statistics, 2) General Search, 3) Search by Gene Ontology, 4) Search by KEGG Pathways. Under the ‘EST Markers’ and ‘EST-SSR Markers’ search utilities, users can easily get the results by entering one of the following keywords: gene product/sequence description, UniProtKB name, gene ontology accession, gene ontology description, pathway name, KEGG pathway Id or KEGG enzyme Id. Keywords are sensitive to spelling mistakes but they are not case-sensitive. For each one of these parameters, an example is set inside the text box below.

For the Mitochondrial drop-down menu, searches are divided into three separate pages (JBrowse, Mitochondrial-SSR and Validated Mitochondrial-SSR). Under the ‘Mitochondrial-SSR’ search utility, users can easily get the results by entering one of the following keywords: gene product, repeat sequence, repeat type or primer Id. Meanwhile, the ‘Validated Mitochondrial-SSR’ page provides users with all information about the *in vitro* validated mtSSR primers/markers.

For the microRNA drop-down menu, search utilities are divided into two separate pages including: 1) JBrowse, 2) Predicted microRNA-targets and markers. Under the ‘JBrowse’ search page, all predicted microRNA-targets and markers were visualized using the JBrowse viewer to display their coordinates within the *V. faba* RefTrans V2. While, in the ‘Predicted microRNA-targets and markers’ search page users can obtain the results by selecting interest microRNA family name/id from a drop-down menu (required) and entering one of the following keywords: *V. faba* target EST name/Id, protein symbol or EST description/gene product (optional).

For the molecular maps drop-down menu, search utilities are presented into two separate pages includes: 1) genetic maps, 2) KEGG pathway-based maps. Under genetic maps page utility, *Vf*ODB provides users with one-page dual-style search feasibility to obtain their results. In the first style, *V. faba* Genetic Maps can be searched conveniently even by making a selection of interest map name or marker type from drop-down menus or by entering one of the following keywords: marker name or linkage group number. While, in the second style, users can simply explore the map of interest among sections that collecting all previously developed genetic maps in *V. faba* and their basic information. In addition, users can deeply visualize and explore the map of interest using ‘Map Browse’ hyperlink in a separate interactive page. Meanwhile, in the KEGG pathway-based maps page, users can basically reach their interest pathway/marker even by making a selection of pathway/marker type from drop-down menus or through scroll-down the interactive table containing all developed *V. faba* KEGG pathway-based maps (functional maps). In addition, users can display and explore our developed markers over the KEGG pathway maps using ‘Browse’ hyperlink in a separate interactive page.

The Germplasm page was implemented initially to provide users with preliminary information about most of the faba bean germplasm available worldwide and listed in the Genesys database as well as the IPK Genebank – Germany. The *Vf*ODB provides users with this information in an easily searchable hyperlinked tabulated style.

**Figure 5.**
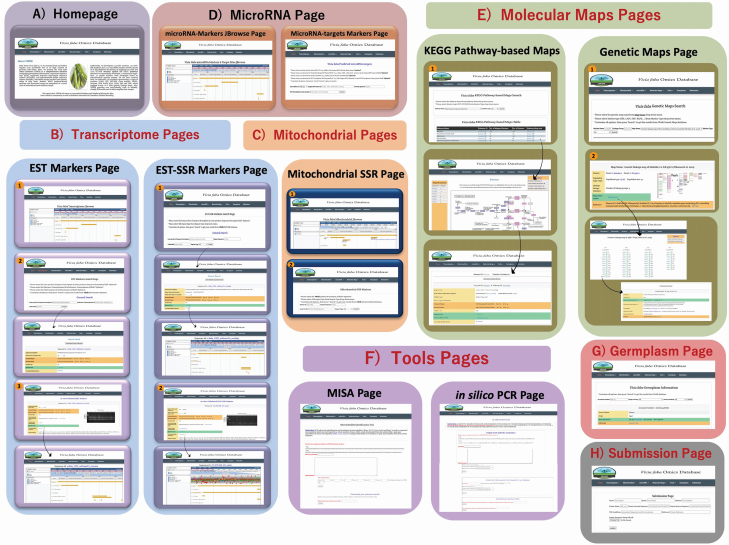
Screenshots of the VfODB database: (A) VfODB homepage, (B) Transcriptome Pages, (C) Mitochondrial Pgges, (D) MicroRNA Page, (E) Molecular Maps Pages, (F) Tools Pages, (G) Germplasm Page and (H) Submission Page.

Almost in all *Vf*ODB searchable pages, users can download/retrieve all obtained or presented results/data sets in a very simple way. The search results of all types of markers are presented in tabulated style containing important related information for each marker (e.g. Primer ID, Repeat Type, Repeat Sequence, Primer Sequence, Primer annealing temp., GC%, Product Length, Gene symbol, etc.). All the above-mentioned *Vf*ODB pages layouts are summarized in Fig. 5.

### *Vf*ODB tools

In the ‘Tools’ section, two powerful sequence analysis tools, MISA (Beier *et al.* 2017) and *in silico* PCR (Kalendar et al. 2011), were configured and implemented into our *Vf*ODB database:

1) The traditional system of developing SSR markers from genomic libraries is smoothly replaced by another modern *in silico* mining approaches. MISA tool is a computational tool used for mining and developing microsatellite markers. MISA can detect perfect microsatellites, as well as compound microsatellites that combine more than one type of simple sequence motifs. Therefore, MISA will act as a useful and efficient helper for researchers interested in the development of SSRs or functional markers in *V. faba*.
2) PCR is a fundamental step in many research fields and is the most significant molecular technique ever been applied. *In silico* PCR or e-PCR, also called virtual-PCR is a computational tool used to mimic theoretically the polymerase chain reaction (PCR) results through simultaneous testing of a single or multiple sets of primer/probe designed to amplify single or multiple target sequences within a given genome or transcriptome sequence and determine all probable PCR products. The *in silico* PCR tool page provides users with two options: 1) using their primer(s) against their interest genome/transcriptome sequences or 2) using their primer(s) against available *V. faba* sequences integrated into our *Vf*ODB database (all NCBI ESTs as well as RefTrans V2). Therefore, implementation of the *in silico* PCR tool in *Vf*ODB is expected to empower users to easily prepare for their experiments with such effective *in silico* modelling approach and in meantime saving their time and effort.

Both MISA and *in silico* PCR tool pages afford the users with availability to retain their previous MISA or *in silico* PCR results and recall it from the *Vf*ODB server within 1 month and download them by just entering the previous job name (ID). Finally, *Vf*ODB presents these tools in a user-friendly graphical user interface supported with cloud processing and database storage availability to facilitate *in silico* mining of microsatellite markers according to the user’s preferences.

The ‘Submission’ page, allows users to easily submit their novel validated markers as well as their amplification conditions in a very straightforward manner and make them freely available.

### *Vf*ODB statistics

**Figure 6.**
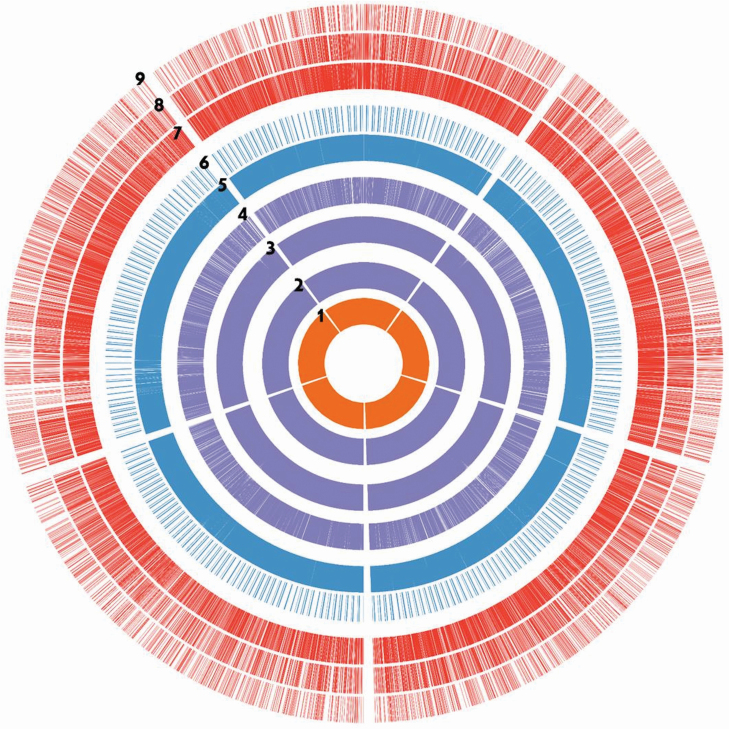
Statistics layout of the *V. faba* Ref-transcriptome mining-based markers. Items order from the inner to outer of circle: 1—total no. of EST sequence we used in analysis; 2—the distribute of designed EST primers; 3—distribute of EST primers with GO ontology; 4—distribute of EST primers assigned with enzymes; 5—distribute of predicted microRNA; 6—distribute of microRNA primers; 7—distribute of designed EST-SSR primers; 8—distribute of EST-SSR primers with GO ontology; and 9—distribute of EST-SSR primers assigned with enzymes.

**Figure 7.**
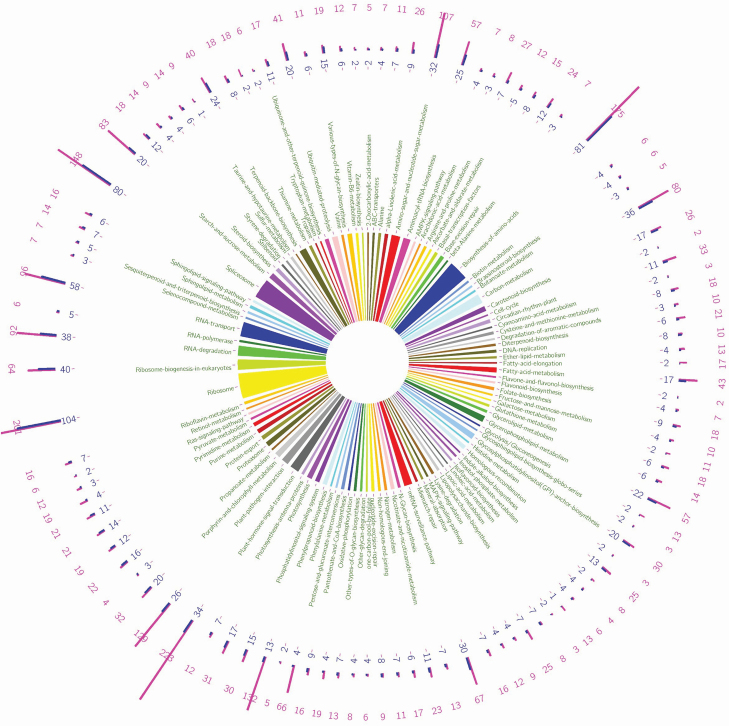
Statistics layout of the *V. faba* KEGG pathway-based maps (Functional Maps). Pathways assigned using the designed SSR primers. The middle part of the figure shows the pathways name. The outer part of the figure reveals the histogram, number of enzymes and number of designed primers (EST or EST-SSR) in each pathway (where the blue colour refers to the number of enzymes in the pathway and the purple colour refers to the number of designed primers in each pathway).

In this study, through the bio-data mining analysis we identified the following:

1) For *V. faba* mitochondrial genome mining, 46 SSR motifs were identified with a high prevalence of mono-nucleotide repeats (26 mtSSR motifs) and lowest prevalence of hexa-nucleotide repeats (2 mtSSR motifs). While, the tri-nucleotide and tetra-nucleotide mtSSR motifs were absent. Among the 46 identified mtSSR motifs, 5 genic-mtSSRs and 41 non-genic mtSSRs were characterized. Finally, 5 genic-mtSSR markers and 35 non-genic mtSSR markers were developed.
2) For *V. faba* Ref-transcriptome mining, 37 378 sequences were analysed to identify and develop EST, EST-SSR and microRNA-target markers. We identified 12 172 SSR motifs and 21 236 microRNA-targets. Thereafter, we successfully developed 31 535 EST, 9071 EST-SSR and 3023 microRNA-target markers with the aid of primer3 software (Untergasser *et al.* 2012). Among the EST developed markers, we identified the Gene-ontology of 17 081 EST markers and assigned 7940 EST markers to a certain enzyme. Meanwhile, among the developed EST-SSR markers, we identified the Gene-ontology of 5217 EST-SSR markers and assigned 2282 EST-SSR markers to a certain enzyme (Fig. 6).
3) For the KEGG pathway-based maps (Functional Maps) development, by mapping of 7940 EST and 2282 EST-SSR markers against the KEGG pathways database we successfully developed 107 KEGG pathway-based maps (Fig. 7).

On the other side, regarding the genetic maps data curation, we retrieved a total of 3461 markers representing 12 types of markers (CAPS, EST, EST-SSR, Gene marker, INDEL, Isozyme, ISSR, RAPD, SCAR, RGA, SNP and SSR) mapped across 18 genetic linkage maps (Table 1a). Among this number, we successfully annotated 889 markers not previously disclosed.

**Table 1.** Summary of the whole analysed data in *Vf*ODB database across the two main data categories.

a)													
	SNP	SSR	RAPD	CAPS	EST-SSR	INDEL	Gene marker	Isozyme	EST	RGA	SCAR	ISSR	Total
No. of markers	2922	136	122	111	74	32	31	16	7	4	3	3	3461
No. of maps	11	6	6	2	2	3	3	5	1	1	2	1	18
b)													
	Total no. of sequences examined			No. of identified motifs/targets		No. of designed primers		No. of sequences assigned gene ontology			No. of sequences assigned enzyme		
EST	37 378			–		31 535		17 081			7940		
EST-SSR				12 172		9071		5217			2282		
microRNA				21 236		3023							

The statistics of the two main data categories (bio-data mining and curation) and their subclasses analysed within the *Vf*ODB database (e.g. no. of tested sequences, no. of identified motifs/targets, no. of developed primers/markers, no. of curated markers per marker-type, etc.) are summarized in Table 1b.

## Discussion and Conclusions

Faba bean is a popular legume crop worldwide because of its rich content of nutrients for human and as animal consumptions. Despite this clear economic and nutritional importance, no public database of the faba bean genomic resources is currently available worldwide. Because of the significance of faba bean and the rapid development of bio-data mining and bioinformatics tools, an online ‘Omics’ hub in faba bean named the *Vf*ODB was constructed. To our knowledge, the *Vf*ODB is the first public species-specific repository with such variability in molecular marker types and maps. It includes functional markers types (such; ESTs, EST-SSRs, genic-mtSSRs, genic-SNPs, microRNA-targets, RGA and gene markers) as well as non-functional marker types (such; non-genic mtSSRs, CAPS, INDEL, Isozyme, ISSR, RAPDs, SCARs, non-genic SNPs and non-genic SSRs markers). Also, the *Vf*ODB is expected to stand as a fully functional hub with different Omics applications in faba bean. Moreover, the *Vf*ODB hub provides satisfactory help material for users to facilitate the use of first-time visitors. These different kinds of functionality can allow researchers to address the roles of developed functional markers on deeper levels and may provide answers to many scientific questions.

The *Vf*ODB database will regularly be updated with newly released genomic, transcriptomics and literature resources. Furthermore, the hub design and tools will be regularly improved, refined and supported. For example, currently, we are aiming to feed the *Vf*ODB database with new *V. faba* transcriptomes developed in our laboratory to address the gene-expression profiles under different abiotic stresses. Also, we aim to link and list all original research works already published or will be released in future especially related to the molecular genetic studies, molecular breeding, genetic mapping, etc., on *V. faba.*

Overall, we believe that the *Vf*ODB database will act as a cornerstone for faba bean research. Also, it will represent great interest to faba bean scientists with different interests including genetic diversity, population genetics, genome mapping, genome evolution, gene-expression profiling, species identification or targeted trait improvement.

## Data Availability

*Vf*ODB is an online free access database initiative available in the following link: http://vfodb.easyomics.org/.

## Notes

**Phenome, Genome & Environment. Chief Editor:** Colleen Doherty
